# Magnetic Resonance Imaging-Based Assessment of Bone Quality Using Vertebral Bone Quality (VBQ) Scores in Spine Surgery—A Critical Assessment and Narrative Review

**DOI:** 10.3390/jcm14186477

**Published:** 2025-09-14

**Authors:** Adeesya Gausper, Wende N. Gibbs, Benjamin D. Elder, Justin K. Scheer, Tiffany G. Perry, Suhas K. Etigunta, Andy M. Liu, Alexander Tuchman, Corey T. Walker

**Affiliations:** 1 Department of Neurosurgery, Cedars-Sinai Medical Center, Los Angeles, CA 90048, USA; 2 Department of Neuroradiology, Barrow Neurological Institute, Phoenix, AZ 85013, USA; 3 Department of Neurosurgery, Mayo Clinic, Rochester, MN 55905, USA; 4 Department of Orthopedic Surgery, Cedars-Sinai Medical Center, Los Angeles, CA 90048, USA

**Keywords:** vertebral bone quality, bone health, fragility fracture, instrumentation failure, subsidence

## Abstract

Bone health is a key determinant of success in spine surgery, making preoperative assessment of bone quality essential to optimal surgical risk stratification. Magnetic resonance imaging (MRI)-based vertebral bone quality (VBQ) score offers a novel approach to assess bone health in spine surgery candidates. The ability of MRI to assess bone quality without exposure to ionizing radiation makes it a potentially advantageous alternative to other traditional measures of bone density. VBQ has additionally shown potential to predict adverse outcomes, such as fragility fractures, instrumentation failure, subsidence and proximal junctional kyphosis. Variations of VBQ, such as endplate bone quality, S1 VBQ, and cervical VBQ, provide targeted insights at specific anatomical regions and potentially enhance the predictive accuracy of VBQ. However, clinical application of VBQ is limited by variability in MRI systems, patient-specific factors, and lack of standardized threshold values. This review aims to critically evaluate VBQ scores as an opportunistic, MRI-based assessment of bone health and its potential role in predicting surgical outcomes. While VBQ may provide some valuable insights into bone health, its role in preoperative risk assessment likely remains supplementary and requires further research to establish clinical validity and optimal cutoffs.

## 1. Introduction

Bone health is crucial for the success and durability of spine surgery, making it a key perioperative consideration. Low bone mineral density (BMD) is a well-known risk factor for adverse surgical outcomes, including instrumentation failure, pseudoarthrosis, proximal junctional kyphosis (PJK), and proximal junctional failure (PJF) [1]. Given these risks, preoperative bone health assessment is essential for optimizing surgical planning.

Dual-energy X-ray absorptiometry (DEXA) is the gold standard for evaluating bone health but consistently fails to fully capture vertebral integrity [2,3]. Nearly half of fragility fractures occur in patients with non-osteoporotic DEXA results [4,5,6,7]. Data suggests DEXA T-scores may not reliably reflect bone health in patients, particularly those with spinal deformities or degenerative changes [8,9]. In contrast, computed tomography (CT) provides microstructural detail, and Hounsfield unit (HU) measurements have been explored as an alternative to DEXA [10]. However, ionizing radiation limits its role in serial monitoring.

Magnetic resonance imaging (MRI)-based vertebral bone quality (VBQ) scores offer a novel approach to bone assessment. VBQ quantifies trabecular marrow composition on T1-weighted sequences, where fatty infiltration serves as a surrogate marker of osteoporosis and indirectly reflects bone density [11]. Since MRI is routinely obtained in spine patients, VBQ offers an “opportunistic” method to assess bone health and stratify risk for complications including instrumentation failure, cage subsidence, and proximal junctional kyphosis. VBQ can be measured regionally (cervical/lumbar), locally (single level, e.g., S1), or focally (endplates) to provide different insights.

Despite a growing body of literature, how VBQ should be incorporated into spine surgery practice remains unclear. This review highlights current research on MRI-based VBQ and explores its potential for predicting surgical outcomes. It is our hypothesis that significant work is needed to establish its clinical utility.

## 2. Materials and Methods

A PubMed/MEDLINE search was conducted through December 2024 using the terms “Vertebral Bone Quality Score” and “VBQ.” Because VBQ is a relatively new imaging metric, these terms reliably captured the available literature. Titles and abstracts were screened, and full texts of relevant articles were reviewed. Reference lists of included studies were examined to identify additional relevant articles. Titles and abstracts included were screened independently by two reviewers, with disagreements resolved by consensus. Included studies spanned a wide range of populations, from adolescents with idiopathic scoliosis to adults and elderly patients with degenerative disease, deformity, fragility fractures, or metastatic tumors. VBQ was investigated both in the context of instrumentation-related outcomes and in broader clinical contexts such as sarcopenia and adolescent bone health evaluation.

Eligibility criteria included original research, reviews, and clinical reports on VBQ. Non-English articles, conference abstracts without full texts, and unrelated studies were excluded. This is a narrative review and therefore may be subject to selection bias. The identified studies were synthesized to provide an overview of VBQ and its clinical significance.

## 3. Using MRI Signal Intensity to Measure Bone Quality

Bone marrow contains fat and water, both of which influence MRI signal intensity (SI). As bone loss occurs, mineralized trabeculae are resorbed and replaced by adipose tissue. Because fat appears hyperintense on T1-weighted sequences, increased marrow fat infiltration results in higher SI [12]. VBQ therefore serves as an indirect but clinically relevant marker of osteoporosis, as greater marrow fat content reflects decreased bone density and diminished trabecular integrity. Although MRI does not provide a direct measurement of bone density in the same manner as CT or DEXA, several studies have noted its relevance in measuring bone health. MRI’s ability to assess changes in vertebral body structure, including trabecular marrow composition, underlies its potential utility for opportunistic evaluation of bone health [11].

### 3.1. Vertebral Bone Quality Calculation

**Figure 1:**
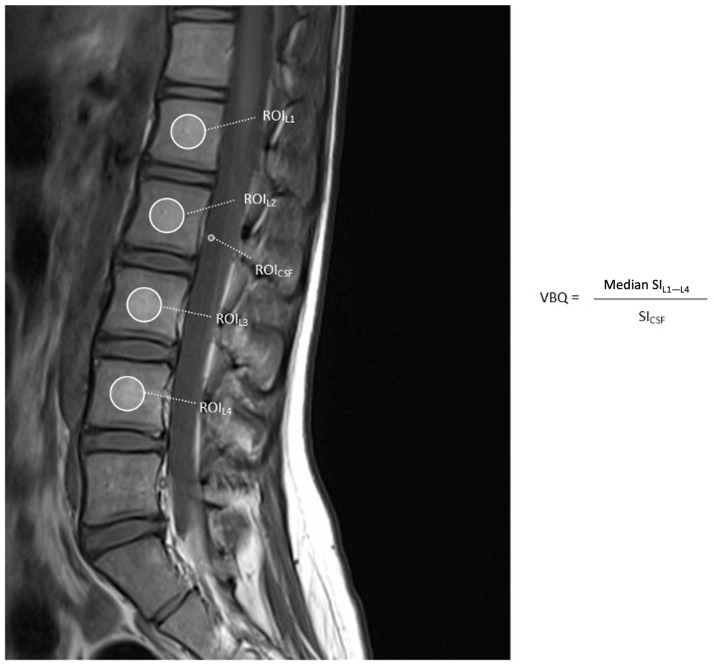
Non-contrast-enhanced T1-weighted MRI of the lumbar spine showing regions of interest (ROIs) used to calculate conventional L1–L4 VBQ score. SI = signal intensity; CSF = cerebrospinal fluid.

Measurement of VBQ was originally described by Ehresman et al. in 2019 [13]. VBQ scores are derived from routine T1-weighted MRI sequences without the need for specialized acquisition protocols. VBQ score is calculated by taking the median SI at a region of interest (ROI) placed over the L1–L4 vertebral bodies, dividing this value by the SI of cerebrospinal fluid (CSF) at L3, which serves as an internal reference to control for variations in scanner hardware and sequence parameters (Figure 1). While differences in field strength, coil design, and acquisition parameters may influence absolute SI values, use of CSF as a reference point improves standardization of VBQ scores across MRI systems.

VBQ = SI_L1–L4_/SI_CSF_ (1)

Increased SI is indicative of higher marrow fat content, reflecting bone loss and reduced trabecular density [14]. Higher VBQ generally indicates lower bone density. This method of VBQ calculation is the most applied method in studies of bone health and surgical outcomes.

### 3.2. Endplate Bone Quality

**Figure 2:**
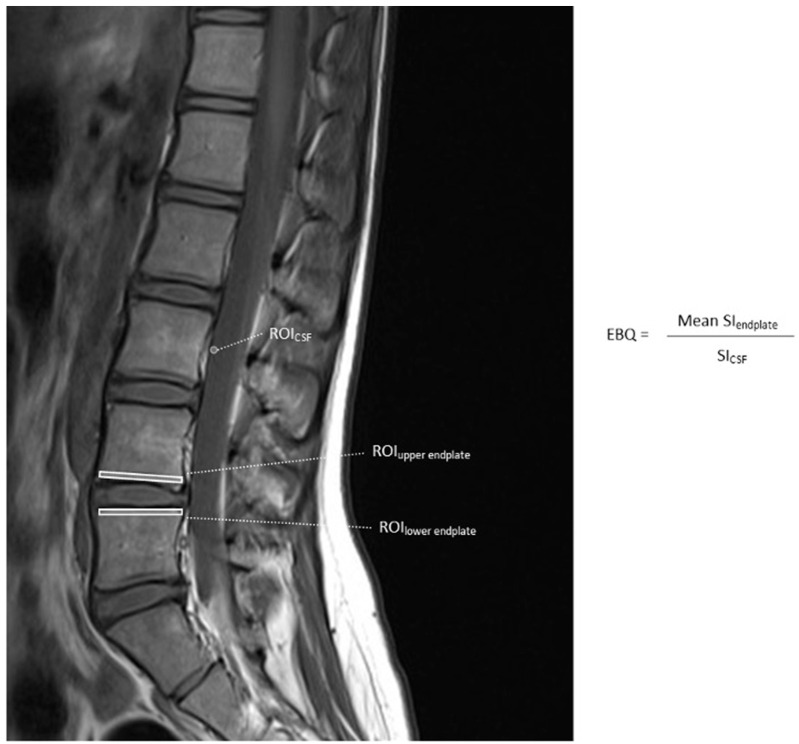
Non-contrast-enhanced T1-weighted MRI of the lumbar spine showing ROIs used to calculate L1–L4 vertebral bone quality (EBQ) score.

Studies have modified conventional VBQ measurements to enhance its predictive value for spine surgery patients. Endplate bone quality (EBQ) reportedly provides a direct assessment of VBQ at the bone–implant interface. In patients undergoing interbody arthrodesis, this ROI at the interface between implants and endplates is critical for achieving arthrodesis and avoiding subsidence. EBQ is measured by averaging the SI at the vertebral subchondral bones adjacent to the endplates and dividing this value by the SI of CSF (Figure 2) [15]. The amount of endplate that should be incorporated into the measurement is not yet determined.

### 3.3. S1 VBQ

**Figure 3:**
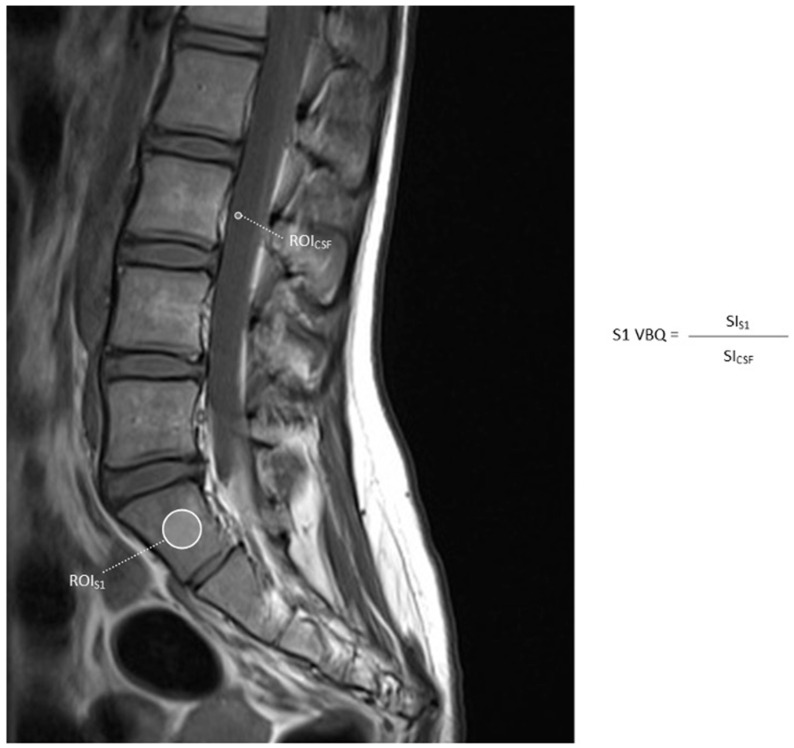
Non-contrast-enhanced T1-weighted MRI of the lumbosacral spine showing ROI used to calculate S1-VBQ score.

Bone density at the S1 region is important for sacropelvic fixation in spinal deformity correction. S1 VBQ may also be helpful in patients with prior instrumentation preventing traditional VBQ measurements. The S1 VBQ is measured by placing the ROI over the S1 vertebra and dividing by the SI of CSF (Figure 3) [16].

## 4. VBQ Evaluation of Osteoporosis

MRI’s ability to estimate the degree of fatty infiltration of trabecular bone marrow, which correlates with bone fragility, positions it as an emerging tool for evaluation of osteoporosis [17]. Multiple studies have found the VBQ to be correlated with DEXA T-score and other bone density metrics, demonstrating moderate predictive value for bone health [18,19,20,21] (Table 1). However, research has also noted that CT-based measurement may have stronger correlation to DEXA T-score compared to VBQ [18,22,23,24]. In addition to VBQ, other MRI-derived measurements are being explored as alternative assessments of bone quality, though access and reliability measures have limited their widespread adoption [17,25]. As such, MRI has been limited in its role for osteoporosis diagnosis. Perhaps using DEXA as the benchmark for comparison may not be the best target, and clinical outcome-driven analysis should be applied instead, as shown below for other spine outcomes.

## 5. Factors That Influence VBQ

### 5.1. Degenerative Spine and Scoliosis

VBQ primarily evaluates SI within trabecular bone, whereas DEXA T-scores merely reflect an average across the anatomy of interest without differentiating between cortical and trabecular bone. VBQ scores may be less prone to influence by spinal deformity or degenerative conditions compared to T-scores. Zheng et al. evaluated conventional and modified VBQ in patients with degenerative lumbar scoliosis and found no significant difference in single-level VBQ scores between patients with moderate and severe scoliosis, indicating reliability in patients with spinal deformity [30].

Ding et al. found that in patients with lumbar spinal stenosis or disc herniation, EBQ was negatively correlated with Pfirrmann disc degeneration grades, but VBQ was not [31]. These findings suggest that disc degeneration primarily affects the endplate, and there is some overlap in the measurements at the disc endplate itself that supports this correlation. VBQ may provide great insight into patients’ bone health overall, which may be potentially more helpful for assessing fracture, kyphosis and pseudarthrosis risks, but EBQ seems most affected by endplate changes seen in degenerative disease.

Chen et al. compared DEXA, VBQ, and CT HU in diffuse idiopathic skeletal hyperostosis (DISH) patients, finding that VBQ and HU values showed no significant differences between groups and effectively distinguished normal BMD from osteopenia and osteoporosis [32].

### 5.2. Limitations in VBQ

Changes in marrow composition due to aging or metabolic conditions, which increase fat infiltration and affect SI, may skew VBQ. Aynaszyan et al. found that hyperlipidemic patients had elevated VBQ, nearing those of osteopenic patients [33]. Li et al. also found that hyperlipidemia may falsely elevate VBQ [20].

VBQ may also be influenced by sex and age. Wang et al. found VBQ more effective at detecting poor bone quality in females than males, though optimal thresholds were similar [34]. Liu et al. observed mean VBQ scores increasing with age, with most patients over 50 having scores > 3.0, reflecting age-related fat infiltration and bone demineralization [35].

Optimal VBQ cutoff values for osteoporosis and osteopenia vary widely, ranging from 2.5 to 14.3 (Table 1), limiting clinical applicability. Similar inconsistencies exist in other MRI-based assessments, such as EBQ values for cage subsidence detection (2.7 to 5.1 (Table 2)), highlighting the need for standardized thresholds.

### 5.3. Interrater and Inter-Device Reliability

The clinical utility of VBQ is dependent on its reliability and consistency across observers. Schilling et al. found VBQ to have good intra- and inter-rater reliability across observers at different levels of training and specialties (ICC = 0.667–0.957) [51]. Mierke et al. similarly found excellent intra- and inter-observer reliability across orthopedic surgery residents [52].

However, device variability remains a significant concern. MRI scanning is complex and based on significant physics. Different machine manufacturers, magnetic field strengths, imaging protocols and echo times vary greatly, which severely undermines the consistency of VBQ. Lin et al. compared VBQ scores using T1-weighted MR with 1.5T and 3.0T field strengths and found that 1.5T MRI VBQ was significantly higher compared to that from 3.0T MRIs [53]. Both 1.5T and 3.0T VBQ were effective in classifying osteoporosis, though 1.5 T VBQ had slightly better predictive performance. Conversely, Pu et al. found no significant difference between VBQ at 1.5T and 3.0T field strengths and with different MRI machines [29].

Roch et al. investigated VBQ calculated using multiple MRI sequences (T1, T2, and STIR) and found that T1 and T2 VBQs were significantly higher in osteoporotic and osteopenic patients but STIR sequence scores were not. Combining VBQ across all three sequences had greater predictive value for osteoporosis than any single sequence alone, although it remains unclear how T2 sequences, which are high for fat and water, and STIR, which is a fat saturation technique, would provide added information when amalgamated [54]. These studies suggest that VBQ can vary across different MRI systems, and standardization of imaging protocols ubiquitously is impossible. Unlike CT HU, VBQ represents a relative rather than an absolute metric, and therefore, variations in relative SI likely will impact measurements and the generalizability of the findings from different imaging locations.

## 6. Predicting Surgical Complications Using VBQ

### 6.1. Cage Subsidence Following TLIF

Interbody subsidence refers to implant settling into adjacent vertebrae, leading to loss of intervertebral height and increased pseudarthrosis risk. It is strongly associated with poor bone quality, where weak vertebrae or endplates cannot support the load transmitted through the cage.

In transforaminal lumbar interbody fusion (TLIF), Soliman et al. and Hu et al. found significantly higher VBQ in patients with subsidence [36,37]. Multivariate analysis identified VBQ as an independent predictor, with Hu et al. reporting 85.6% accuracy at a cutoff of 3.280 [37].

VBQ’s correlation with other bone quality measures has also been studied. Ai et al. found a moderate correlation between VBQ and quantitative CT (QCT) (r = −0.426), with QCT being slightly more accurate in predicting subsidence (AUC VBQ 0.825 vs. QCT 0.857) [38]. Their follow-up study confirmed significantly higher VBQ and EBQ in patients with subsidence, with EBQ having better predictive value (AUC 0.829 vs. 0.799) [40]. Chen et al. also found EBQ was higher in subsidence cases (5.0 vs. 4.3), with EBQ showing 82.0% accuracy at a threshold of 4.7 [39].

### 6.2. Cage Subsidence Following OLIF and LLIF

While TLIF is performed through a posterior transforaminal approach, anterolateral lumbar approaches such as oblique lumbar interbody fusion (OLIF) and lateral lumbar interbody fusion (LLIF) allow placement of larger grafts with less posterior soft tissue and neural element disruption. While subsidence rates are lower than in TLIF, graft settling, when it occurs, leads to loss of indirect decompression and pseudarthrosis, making preoperative risk assessment valuable.

Huang et al. found higher VBQ to be significantly associated with increased subsidence risk after OLIF, with VBQ outperforming DEXA (83.9% vs. 69.5%) [41]. Pu et al. compared CT HU and VBQ, finding segmental L4-L5 VBQ more predictive of subsidence, with combined segmental VBQ and HU scores offering the highest accuracy (86.5%) [42].

Ran et al. reported significantly higher EBQ in subsidence cases (3.48 vs. 2.31), with an 81.1% AUC at a threshold of 2.3 [43]. Zheng et al. found that VBQ, mean VBQ, and EBQ were all higher in patients with subsidence [44]. In standalone OLIF, EBQ was the strongest predictor (OR = 13.656), while mean VBQ was most predictive in OLIF with posterior fixation (OR = 8.301). These findings suggest site-specific assessments may enhance surgical planning over whole-region bone health measurements.

Jones et al. found that both EBQ and VBQ were higher in patients with cage subsidence following standalone LLIF [15]. EBQ was an independent predictor for severe cage subsidence after adjusting for Modic changes and QCT-volumetric BMD. EBQ had slightly better predictive value for subsidence than VBQ, with a predictive accuracy of 61% using a cutoff score of 5.1.

### 6.3. Pedicle Screw Loosening

Pedicle screws depend on the bone’s strength for stability, and poor bone quality can lead to loosening. Several studies highlight the potential of VBQ to identify at-risk patients. Chen et al. [45] and Li et al. [48] found significantly higher VBQ in patients with screw loosening following posterior lumbar interbody fusion (PLIF). VBQ was an independent predictor, slightly outperforming CT HU (72.0% vs. 70.2%).

Gao et al. similarly reported higher VBQ in patients with screw loosening following thoracolumbar fusion (3.61 vs. 2.86), with a predictive accuracy of 74.4% at an optimal cutoff of 3.055 [47]. Li et al. found significantly higher mean S1 VBQ in patients with loosening after adult deformity surgery and a predictive accuracy of 74.6% at a threshold of 3.175 (3.31 vs. 3.01) [46].

### 6.4. Proximal Junctional Kyphosis

PJK is a significant complication following long-segment spinal fusion, leading to pain, neurologic deficits, and need for revision surgery. Kuo et al. found that patients who developed proximal junctional failure (PJF) had significantly higher VBQ (3.13 vs. 2.46) [49]. VBQ was an independent predictor of PJK on multivariate analysis and demonstrated a predictive accuracy of 94.3% on ROC analysis. Deng et al. found a significantly higher S1 VBQ in patients who developed PJK (3.58 vs. 3.08) [50]. These studies suggest that VBQ may be an additive adjunct in pre-operative PJK/PJF risk assessment but, again, it remains unclear which measure or cutoff to use in this population.

### 6.5. Reoperation and Adjacent Segment Disease

Kuo et al. found that higher VBQ was linked to increased risk of adjacent segment disease (ASD) following lumbar interbody fusion [55]. The AUC for surgical ASD was 0.934, with a cutoff of 2.91. Ehresman et al. found that patients requiring reoperation after lumbar fusion had significantly higher preoperative VBQ (3.29 vs. 2.92) [56]. A VBQ cutoff of 3.0 identified 70% of reoperation patients. Notably, DEXA T-scores did not differ significantly between patients who did and did not require reoperation.

Ramos et al. showed that combining VBQ with fusion risk score (FRS) predicted 90-day reoperation with an AUC of 0.808, compared to FRS alone (AUC 0.783) [57,58]. A VBQ higher than 2.6 was associated with more than double the risk of complications. However, the VBQ/FRS combination had limited predictive ability for readmissions and other outcomes, indicating that VBQ is not universally predictive. While the exact mechanism remains unclear, patients with osteosarcopenia may be at higher risk of adjacent segment degeneration and reoperation. Future research should clarify the mechanisms behind VBQ’s correlation with adverse outcomes.

## 7. Sarcopenia

Sarcopenia refers to the loss of muscle tissue often seen in older adults. This syndrome can lead to increased risk of falls and fractures, and VBQ may be a useful tool in identifying at-risk patients. Li et al. found that patients with VBQ scores > 3.0 had smaller cross-sectional areas (CSA) of the paravertebral muscles and a higher degree of fat infiltration (FI), highlighting the link between poor bone quality and sarcopenia [59]. On multivariate analysis, CSA and FI correlated more to VBQ than DEXA BMD, suggesting that VBQ may be an imaging biomarker of sarcopenia. Moser et al. found that higher VBQ was significantly correlated with smaller psoas CSA at L3 in men [60]. Interestingly, this correlation was not observed in women in their study, which may reflect hormonally differentiated patterns of musculoskeletal degeneration between sexes.

## 8. Fragility Fractures

Osteoporotic vertebral compression fractures (VCF) are a significant concern in patients with low bone density. VBQ’s predictive value for VCF is shown (Table 3). Ehresman et al. found higher VBQ in patients with new fragility fractures (3.50 vs. 3.01) [61]. VBQ was superior to DEXA scores in predicting fracture risk. Yin et al. also found higher VBQ in VCF patients (4.21 vs. 3.84) with 73.54% predictive accuracy at a cutoff of 3.70 [62]. Li et al. reported higher VBQ in VCF patients (4.0 vs. 3.5), with predictive accuracy of 0.815 [63,64].

Wang et al. found that increased S1 and L1–L4 VBQ and decreased CT HU values predicted OVCF [65]. Combining these scores improved diagnostic accuracy (AUC S1 VBQ + HU 0.862). Yu et al. identified VBQ as a risk factor for vertebral recompression after kyphoplasty, with adjacent-to-injured VBQ ratio being the most significant predictor [66]. However, some studies show limitations in VBQ’s ability to predict fragility fractures. Zhang et al. compared CT HU, MRI VBQ, and DEXA BMD in predicting thoracolumbar fractures, with HU having the highest AUC (0.863) [67]. VBQ had the lowest (AUC 0.602). Wang et al. found T2 VBQ scores (AUC 0.82) more effective than T1 VBQ (AUC 0.72) in predicting fractures [68].

**Table 3:** MRI VBQ threshold values for predicting fracture.

Publication	Mean VBQ Fracture vs. No Fracture	Threshold Value	Sensitivity	Specificity	AUC	Odds Ratio (OR)
*Fragility fracture*						
Li et al., 2022 [63]	**4.0 vs. 3.5**	--	--	--	--	**2.58**
Yin et al., 2023 [62]	**4.21 vs. 3.84**	3.72	81.3%	45.1%	0.6717	**1.496**
Ehresman et al., 2021 [61]	**3.50 vs. 3.01**	--	--	--	--	**2.40**
Li et al., 2023 [64]	**3.58 vs. 2.88**	3.22	68.8%	84.4%	0.815	--
Li et al., 2023 [64]	Single level L1 VBQ **3.60 vs. 2.95**	3.16	81.7%	68.8%	0.817	--
Wang et al., 2024 [65]	**3.58 vs. 3.13**	3.32	83%	72%	0.799	**3.07**
Wang et al., 2024 [65]	S1 VBQ **3.73 vs. 3.11**	3.40	81%	72%	0.806	**3.33**
Zhang et al., 2023 [67]	**3.50 vs. 3.27**	3.37	53.85%	74.36%	0.602	--
*Pathologic fracture*						
Ehresman et al., 2019 [13]	**3.26 vs. 2.48**	3.0	75.0%	85.7%	0.80	**3.051**
Pennington et al., 2023 [69]	2.70 vs. 2.49	--	--	--	--	--
Pennington et al., 2023 [69]	VBQ at tumor level ±1 2.77 vs. 2.53	--	--	--	--	--
Pennington et al., 2023 [69]	VBQ at level above and below tumor 3.21 vs. 3.74	--	--	--	--	--

**Bold denotes significant difference or significant value**.

### Pathologic Fracture

Metastatic spinal tumors can weaken the structural integrity of affected vertebra, leading to pathologic fractures. Ehresman et al. compared VBQ with the Spinal Instability Neoplastic Score (SINS) in patients with spinal metastases to predict pathologic fracture [13]. Patients with VCF had significantly higher VBQ than those without (3.26 vs. 2.48, *p* < 0.0001), and both VBQ and SINS were found to be significant predictors of new VCF. In contrast, Pennington et al. found that VBQ did not significantly predict risk of pathologic fracture in patients with spinal metastasis [69]. CT HU score ≤ 132 and SINS score ≥ 7 were both found to be significant predictors of VCF on survival analysis, and only HU independently correlated.

## 9. Cervical VBQ Scores

**Figure 4:**
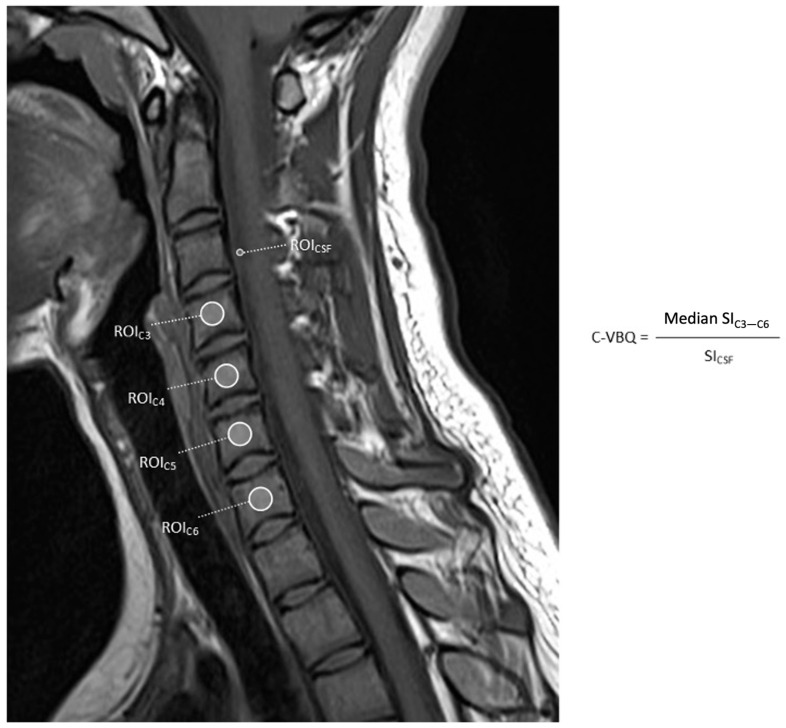
Non-contrast-enhanced T1-weighted MRI of the cervical spine showing ROIs used to calculate C-VBQ.

Conventional VBQ in lumbar vertebrae may not reflect the unique anatomical and biomechanical factors influencing cervical bone quality. Studies have explored cervical VBQ (C-VBQ), which places the ROI in cervical bodies for region-specific evaluation (Table 4). C-VBQ is calculated by taking the median SI of C3–C6 and dividing it by the SI of the C2 CSF (Figure 4) [70].

Razzouk et al. found moderate correlations between cervical, thoracic, and lumbar VBQ scores, with weaker correlation of cervical and lumbar scores (r = 0.324) [71]. Kuo et al. observed a strong correlation between conventional VBQ and C-VBQ scores (r = 0.757) among patients undergoing spine surgery [72]. Aguirre et al. similarly found three C-VBQ methodologies significantly correlated with conventional VBQ (*p* < 0.001) [73].

Oezel et al. found that VBQ values across different cervical levels were highly correlated, but correlations between VBQ and QCT-derived BMD were weak [74], suggesting C-VBQ may not reliably estimate bone quality on its own. Wang et al. found that multiple C-VBQ methodologies significantly correlated with DEXA [75]. The C-VBQ method dividing the median SI of C2–C7 by T1 CSF yielded the highest AUC of 0.727, while combining HU and C-VBQ values produced the highest AUC of 0.786. Huang et al. found a significant difference in cervical VBQ between patients with osteoporosis/osteopenia and those with normal BMD (3.80 vs. 2.99, *p* < 0.001) [76]. There was a strong correlation between C-VBQ and DEXA, and C-VBQ demonstrated 78% accuracy with a cutoff score of 2.905.

**Table 4:** MRI VBQ threshold values for predicting cervical spine surgery complications.

Publication	Procedure	Mean VBQ Complication vs. No Complication	Threshold Value	Sensitivity	Specificity	AUC	Odds Ratio
*Cage subsidence*							
Bernatz et al., 2024 [77]	ACDF	**3.80 vs. 2.40**	3.2	100%	94.1%	0.99	--
Li et al., 2024 [78]	ACCF	**3.75 vs. 3.20**	3.445	69.6%	85.2%	0.810	**13.563**
Soliman et al., 2023 [70]	ACDF	**2.83 vs. 2.22**	--	--	--	--	**1.85**
Li et al., 2024 [79]	ACDF	**3.33 vs. 2.36**	2.92	78.9%	85.7%	0.892	--
Li et al., 2024 [79]	ACDF	C-EBQ **2.59 vs. 1.81**	2.12	84.8%	89.8%	0.937	**5.700**
Wang et al., 2024 [80]	ACDF	**2.94 vs. 2.33**	2.68	72.7%	82.1%	0.785	**1.823**
*Distal junctional kyphosis*							
Aguirre et al., 2024 [81]	Posterior cervical fusion	**2.97 vs. 2.26**	2.66	84.2%	81.1%	0.886	**1.46**

Bold denotes significant difference or significant value; ACDF = anterior cervical discectomy and fusion; ACCF = anterior cervical corpectomy and fusion.

### Prediction of Cervical Surgery Outcomes

Li et al. found that patients who experienced titanium mesh cage subsidence after anterior cervical corpectomy and fusion (ACCF) had significantly higher VBQ (3.75 vs. 3.20) [78]. Multivariate analysis identified VBQ as the only independent predictor of subsidence, with an 81.0% accuracy using a cutoff of 3.445. There was a significant positive correlation between VBQ score and subsidence (r = 0.509).

Soliman et al. found that patients with subsidence following anterior cervical discectomy and fusion (ACDF) had significantly higher C-VBQ (2.83 vs. 2.22) [70]. There was a significant negative correlation between C-VBQ and HU, and C-VBQ was the only significant predictor of cage subsidence (OR = 1.85). Wang et al. also reported significantly higher C-VBQ in patients with zero-profile cage subsidence following ACDF compared to those without (2.94 vs. 2.33) [80]. C-VBQ was also found to be a significant predictor of cage subsidence (OR = 1.823). There was a moderate negative correlation between HU and C = VBQ (r = −0.507), and C-VBQ had an AUC of 0.785 using a cutoff value of 2.68. Notably, HU had a better predictive value for subsidence with AUC of 0.826.

Li et al. introduced C-EBQ, calculated by dividing the SI at the endplate by the SI at the C2 CSF [79]. C-EBQ was significantly higher in patients with subsidence following ACDF (2.59 vs. 1.81) with a superior predictive accuracy of 93.7% compared to C-VBQ (89.2%). C-EBQ was the only significant independent predictor of cage subsidence.

Aguirre et al. found C-VBQ was an independent predictor of distal junctional kyphosis following posterior cervical fusion [81]. C-VBQ was significantly higher in patients with DJK (2.97 vs. 2.26), and greater C-VBQ was associated with greater kyphotic angle changes (r^2^ = 0.26). ROC demonstrated a diagnostic accuracy of 88.6% for DJK using a C-VBQ cutoff of 2.66.

These studies highlight C-VBQ’s value in predicting cervical surgery outcomes, offering insight into cervical bone quality, particularly as CT HU measurements show inconsistency across levels [82].

## 10. Adolescent Patients

The effectiveness of VBQ in younger populations is not fully established. Patel et al. investigated VBQ and DEXA Z-scores in adolescents aged 11–21, finding a negative correlation, with females showing slightly higher scores than males [83]. Yang et al. found that adolescents with idiopathic scoliosis and low QCT-derived BMD had significantly higher VBQ scores (3.48 vs. 2.62) [84]. VBQ predicted low BMD with 81% accuracy at a cutoff of 3.18. Ramos et al. found higher VBQ in patients with scoliosis (2.5 vs. 2.1) [85]. These studies suggest VBQ may be useful for evaluating bone health in adolescents, especially as a radiation-free alternative for preoperative assessment.

## 11. Discussion

VBQ studies have received an enormous amount of attention in the merely 5 years since first being applied for spinal conditions. This likely can be attributed to the relative availability of MRI studies in spine surgery patient cohorts and the ease with which retrospective evaluation of VBQ’s correlation to specific clinical outcomes can be executed. Nevertheless, to date, no one has summarized the existing reports or analyzed the pros and cons of this imaging modality for clinical applications.

Based on the current data, there is undoubtedly an imaging biomarker signal that is captured by VBQ. In multiple studies VBQ has demonstrated the ability to detect changes in bone quality, which may prognosticate fracture risk, subsidence, screw pullout, adjacent segment disease, proximal junctional failures and re-operation. There appears to be high inter-rater reliability, with application to all areas of the spine, and MRI is abundantly available. Moreover, focal measurements of the sacrum, endplates or operative segments may help give poignant details specific to the exact procedure to be performed.

There are several distinct limitations of VBQ. Firstly, patients who are unable to have an MRI are not eligible for this evaluation, and there may be specific limitations that can impact SI, including metal artifacts, cement, neoplastic disease and global lipid burden. Sex- and age-related differences may too impact results and need to be further studied. The most important shortcoming is that SI in MRI is a relative value. It is impacted by the MRI scanner used, magnet strength, protocol and parameter settings. Furthermore, some centers elect to use T1 FLAIR sequences (most scanners with 3 Tesla magnets), which undermines the ability to relegate CSF as a reference value for measurements. Therefore, the relativity of VBQ will always affect the standardization of measurements and the generalizability of research findings. This is exemplified by the wide range of threshold cutoffs seen in the various studies reviewed here. This leaves clinicians unable to broadly apply VBQ in their own practice, as it is unclear what the optimal thresholds are for VBQ, segmental VBQ, EBQ and C-VBQ that will provide the highest balanced accuracy. Consequently, it is the authors’ feeling that VBQ should not yet be used alone on a daily basis for spine surgery clinical decision-making. Future work should focus on the development of standardized MRI acquisition protocols to minimize inter-scanner variability. This may involve consensus guidelines for sequence selection, repetition and echo times, and methods to normalize intensity. Establishing normative VBQ ranges across demographic groups, including age and sex, will further strengthen its clinical applicability by providing reference standards for interpretation. Future MRI-based imaging strategies may be developed to better define risk, and advanced computational algorithms may be required to harness the prognostic value of MRI to achieve direct clinical utility. Machine learning can help develop post-processing methods, such as automated normalization across scanner types or correction for field strength differences. This may preserve the opportunistic nature of VBQ while improving reproducibility. Radiomics-based models may also facilitate integration of VBQ with other imaging biomarkers to refine risk stratification. Until that occurs, CT-based measurements of bone quality should prevail as the most informative metrics for evaluating bone health in the clinic.

## 12. Conclusions

MRI-based VBQ assessments are a new tool for assessing bone quality and predicting surgical outcomes in spine surgery. VBQ correlates with traditional bone density measures, with higher scores linked to an increased risk of adverse outcomes. Modifications such as EBQ, S1 VBQ, and C-VBQ provide region-specific assessments that may enhance predictive power.

Despite its potential, VBQ’s broader application is limited by variability across MRI systems, patient-specific factors, and the lack of standardized thresholds. While its clinical utility in preoperative risk stratification remains unclear, it may complement DEXA or CT in cases where they fail to capture bone strength loss. Future research must define serviceable cutoffs to enhance its clinical value to render it clinically beneficial. Combining VBQ with other measures, like HU, could improve its role in surgical decision-making; however, we feel that at the current time, the use of CT HU should remain as the mainstay opportunistic measurement of bone density.

## Figures and Tables

**Table 1:** MRI VBQ threshold values for detection of osteoporosis/osteopenia (OP).

Publication	Mean VBQ OP vs. No OP	Threshold Value	Sensitivity	Specificity	AUC
Ehresman et al., 2020 [19]	**3.31 vs. 2.74**	--	--	--	0.81 *
Kadri et al., 2022 [26]	**3.67 vs. 2.79**	2.95	91%	60%	0.806 *
Salzmann et al., 2022 [18]	**2.6 vs. 2.2**	2.388	74.3%	57%	0.7079 **
Chen et al., 2023 [27]	--	2.83	93%	65.4%	0.818 *
Courtois et al., 2023 [22]	**2.78 vs. 2.55**	2.50	54.7%	51.4%	0.557 *
Huang et al., 2023 [16]	**3.80 vs. 2.85**	--	--	--	--
Huang et al., 2023 [16]	S1 VBQ **3.79 vs.** **2.69**	2.93	--	--	0.82 *
Kim et al., 2023 [28]	**2.7 vs. 2.2**	2.6	58%	90%	0.754 ***
Ozmen et al., 2023 [21]	**3.02 vs. 2.69**	2.7	83.3%	44.3%	0.667 *
Pu et al., 2023 [29]	**3.43 vs. 2.81**	3.05	87.5%	61.8%	0.810 *
Xu et al., 2023 [23]	**15.09 vs. 12.42**	14.324	36.1%	93.9%	0.771 *

**Bold denotes significant difference.** * OP diagnosis based on DEXA T-scores. ** OP diagnosis based on QCT BMD. *** OP diagnosis based on QCT-derived T-score.

**Table 2:** MRI VBQ threshold values for predicting postoperative complications.

Publication	Procedure	Mean VBQ Complication vs. No Complication	Threshold Value	Sensitivity	Specificity	AUC	Odds Ratio (OR)
*Cage subsidence*							
Soliman et al., 2022 [36]	TLIF	**2.9 vs. 2.5**	--	--	--	--	**1.5**
Hu et al., 2022 [37]	TLIF	**3.79 vs. 2.96**	3.28	85.6%	78.6%	0.856	**14.615**
Ai et al., 2023 [38]	TLIF	**3.7 vs. 3.1**	3.5	87.5%	74.5%	0.825	**2.690**
Chen et al., 2023 [39]	TLIF	EBQ **5.0 vs. 4.3**	4.73	76.2%	83.2%	0.820	**1.02**
Ai et al., 2024 [40]	TLIF	**3.7 vs. 3.1**	3.4	84.6%	69.2%	0.799	**4.557**
AI et al., 2024 [40]	TLIF	EBQ **5.0 vs. 4.3**	4.7	76.9%	82.7%	0.829	**5.396**
Huang et al., 2023 [41]	OLIF	**3.83 vs. 2.98**	3.435	69.23%	88.89%	0.839	**23.158**
Pu et al., 2023 [42]	OLIF	Global VBQ **5.10 vs. 3.31** *	4.10	73.3%	83.8%	0.814	--
Pu et al., 2023 [42]	OLIF	Segmental VBQ **5.07 vs. 3.32** **	3.36	80.0	81.1%	0.820	--
Ran et al., 2024 [43]	OLIF	EBQ **3.48 vs. 2.31**	2.318	93.1%	55.9%	0.811	**6.204**
Zheng et al., 2024 [44]	OLIF	**3.21 vs. 2.85**	--	--	--	--	--
Zheng et al., 2024 [44]	OLIF	EBQ 2.97 vs. 2.75	--	--	--	--	--
Zheng et al., 2024 [44]	SA-OLIF	**3.14 vs. 2.82**	2.84	--	--	0.684	--
Zheng et al., 2024 [44]	SA-OLIF	EBQ **2.79 vs. 2.41**	2.60	65.4%	75.0%	0.745	**13.656**
Zheng et al., 2024 [44]	OLIF-PF	**3.32 vs. 2.88**	3.20	--	--	0.757	--
Zheng et al., 2024 [44]	OLIF-PF	EBQ 3.20 vs. 3.02	--	--	--	--	1.721
Jones et al., 2022 [15]	LLIF	**2.67 vs. 2.39**	--	--	--	--	1.79
Jones et al., 2022 [15]	LLIF	EBQ **5.09 vs. 4.31**	5.1	40.0%	84.5%	0.61	**0.80**
*Screw loosening*							
Chen et al., 2022 [45]	PLIF	**3.1 vs. 2.8**	2.87	76.9%	64.8%	0.744	**1.02**
Li et al., 2024 [46]	PLIF	S1 VBQ **3.31 vs. 3.01**	3.175	76.0%	83.3%	0.746	**5.778**
Gao et al., 2024 [47]	Lumbar fusion	**3.61 vs. 2.86**	3.055	81.8%	71.3%	0.774	**3.555**
Li et al., 2023 [48]	Lumbar fusion	**3.28 vs. 2.82**	3.05	65.5%	71.3%	0.720	**3.908**
*Proximal junctional kyphosis*							
Kuo et al., 2023 [49]	Thoracolumbar fusion	**3.13 vs. 2.46**	2.85	88.2%	95.1%	0.943	**1.745**
Deng et al., 2024 [50]	Thoracolumbar fusion	S1 VBQ **3.58 vs. 3.08**	3.205	77.8%	81.4%	0.721	**4.565**

Bold denotes significant difference or significant value; TLIF = transforaminal lumbar interbody fusion; OLIF = oblique lumbar interbody fusion; SA-OLIF = stand-alone OLIF; OLIF-PF = OLIF with posterior internal fixation; LLIF = lateral lumbar interbody fusion; PLIF = posterior lumbar interbody fusion. * Global VBQ defined as median SI of L1-L5 divided by mean SI at L3 CSF space. ** Segmental VBQ defined as mean SI of L4-L5 divided by the mean SI at L3 CSF space.

## Abbreviations

The following abbreviations are used in this manuscript:

BMD	Bone mineral density
PJK	Proximal junctional kyphosis
PJF	Proximal junctional failure
DEXA	Dual-energy X-ray absorptiometry
CT	Computed tomography
HU	Hounsfield unit
MRI	Magnetic resonance imaging
SI	Signal intensity
ROI	Region of interest
CSF	Cerebrospinal fluid
EBQ	Endplate bone quality
OP	Osteoporosis/osteopenia
AUC	Area under the curve
QCT	Quantitative computed tomography
DISH	Diffuse idiopathic skeletal hyperostosis
OR	Odds ratio
TLIF	Transforaminal lumbar interbody fusion
OLIF	Oblique lumbar interbody fusion
SA-OLIF	Standalone oblique lumbar interbody fusion
OLIF-PF	Oblique lumbar interbody fusion with posterior fixation
LLIF	Lateral lumbar interbody fusion
ICC	Intraclass correlation coefficient
VCF	Vertebral compression fracture
ASD	Adjacent segment disease
FRS	Fusion risk score
CSA	Cross-sectional area
FI	Fat infiltration
C-VBQ	Cervical vertebral bone quality
C-EBQ	Cervical endplate bone quality
ACCF	Anterior cervical corpectomy and fusion
ACDF	Anterior cervical discectomy and fusion
DJK	Distal junctional kyphosis
SINS	Spinal instability neoplastic score
